# Preventing Maritime Transfer of Toxigenic *Vibrio cholerae*

**DOI:** 10.3201/eid1810.120676

**Published:** 2012-10

**Authors:** Nicole J. Cohen, Douglas D. Slaten, Nina Marano, Jordan W. Tappero, Michael Wellman, Ryan J. Albert, Vincent R. Hill, David Espey, Thomas Handzel, Ariel Henry, Robert V. Tauxe

**Affiliations:** Centers for Disease Control and Prevention, Atlanta, Georgia, USA (N.J. Cohen, D.D. Slaten, N. Marano, J.W. Tappero, M. Wellman, V.R. Hill, D. Espey, T. Handzel, R.V. Tauxe);; US Environmental Protection Agency, Washington, DC, USA (R.J. Albert);; and Haitian Ministry of Public Health and Population, Port-au-Prince, Haiti (A. Henry)

**Keywords:** Toxigenic, Vibrio cholerae, cholera, Haiti, Caribbean, ballast water, ships, epidemic, bacteria, enteric infections

## Abstract

Organisms, including *Vibrio cholerae*, can be transferred between harbors in the ballast water of ships. Zones in the Caribbean region where distance from shore and water depth meet International Maritime Organization guidelines for ballast water exchange are extremely limited. Use of ballast water treatment systems could mitigate the risk for organism transfer.

Cholera is an acute diarrheal illness caused by toxigenic strains of the bacterium *Vibrio cholerae* serogroups O1 and O139. *V. cholerae*, like other vibrios, is found commonly in marine and estuarine environments, living freely or on surfaces, such as plants and animal shells, and in intestinal contents of marine animals (*1*). *V. cholerae* infection is typically acquired by ingestion of contaminated water or food (*2*).

Ballast water is collected in ships to regulate their stability; the discharge of ballast water can transfer toxigenic *V. cholerae* O1 from one harbor to another (*3*). During 1992, shellfish in Mobile Bay, Alabama, on the US coast of the Gulf of Mexico, were contaminated with an epidemic strain of toxigenic *V. cholerae* O1 from Latin America, although no human illnesses were reported (*4*). *V. cholerae* transfer by cargo ship was documented when the same strain was isolated from ballast and other nonpotable water samples collected from 5 cargo ships from ports in Latin America that arrived in the US Gulf of Mexico (*5*).

To reduce the risk for transfer of invasive species and pathogens between harbors by introduction of contaminated ballast water, the International Maritime Organization (IMO) adopted the International Convention for the Control and Management of Ships’ Ballast Water and Sediments (BWM Convention) on February 13, 2004 (*6*). Regulation D-2 of the convention establishes numeric ballast water discharge standards, to be phased in with a start date of January 1, 2012, that limit the concentration of viable organisms and human pathogens (including toxigenic *V. cholerae*, *Escherichia coli*, and intestinal enterococci). The limit for toxigenic *V. cholerae* is <1 CFU/100 mL or <1 CFU/g (wet weight) zooplankton samples. In the interim, the BWM Convention requires that, whenever possible, ships conduct ballast water exchange >200 nautical miles from the nearest land and in water >200 m deep. If these requirements cannot be met, the exchange should be performed as far from the nearest land as possible, but at a minimum >50 nautical miles from the nearest land and in water >200 m deep. When these requirements cannot be met, areas may be designated where ships can conduct ballast water exchange. Ballast water exchange is based on the principles that 1) organisms from coastal areas will not survive in the open ocean and 2) fewer organisms (including fewer human pathogens) will be taken up in the open ocean, and these will be less likely to adapt to coastal waters.

A cholera epidemic emerged in Haiti in October 2010; lack of safe water and sanitation infrastructure and the devastation caused by the January 2010 earthquake contributed to its spread (*7*). Concerns were raised that cholera could be transferred from Haiti to other countries through contamination of coastal waters by ship ballast water. Ship traffic to Haiti (233 vessel calls in Port-au-Prince in 2008) consists predominantly of cargo vessels with destinations in the United States, other Caribbean islands, and Latin America (*8*). During an assessment of cholera contamination of fresh and marine water sources in Haiti during the epidemic conducted by the US Centers for Disease Control and Prevention (CDC), US Food and Drug Administration, and the Haitian Ministry of Health and Population, water and seafood collected from harbors at Port-au-Prince and St. Marc were tested for viable *V. cholerae* and for the cholera toxin gene (*ctx*A) (*9*). Toxigenic *V. cholerae* O1 serotype Ogawa indistinguishable from the outbreak strain was isolated from seafood samples from Port-au-Prince. Although *V. cholerae* was not isolated from marine water samples, the *ctx*A gene was detected in broth cultures of seawater samples from both harbors, suggesting that harbor waters were contaminated with toxigenic *V. cholerae*.

## The Study

To further evaluate the risk for cholera transfer through ballast water under existing management approaches, we applied the IMO ballast water exchange depth and distance criteria to the Caribbean region. Buffers of 50 and 200 nautical miles were generated on the basis of the Global Self-Consistent, Hierarchical, High-Resolution Shoreline Database (version 2.1; www.ngdc.noaa.gov/mgg/shorelines/gshhs.html) and overlaid on bathymetric data from the ETOPO1 Global Relief Model (www.ngdc.noaa.gov/mgg/global/global.html) (*10*,*11*). We acquired these datasets through the National Oceanic and Atmospheric Administration’s National Geophysical Data Center (www.ngdc.noaa.gov).

Mapping indicates that waters around Haiti where the IMO guidelines can be followed are extremely limited (Figure). To exchange ballast >200 nautical miles from shore in water 200 m deep, ships must travel 280 nautical miles northeast of Haiti (Figure, A) or to the Gulf of Mexico (Figure, B). To exchange ballast at the minimum 50 nautical miles from shore in water >200 m deep, ships must travel >90 nautical miles northeast (Figure, C) or 50 nautical miles south (Figure, D) of Haiti or conduct the exchange in an area <45 nautical miles wide approximately equidistant from Haiti, Cuba, and Jamaica (Figure, E).

**Figure:**
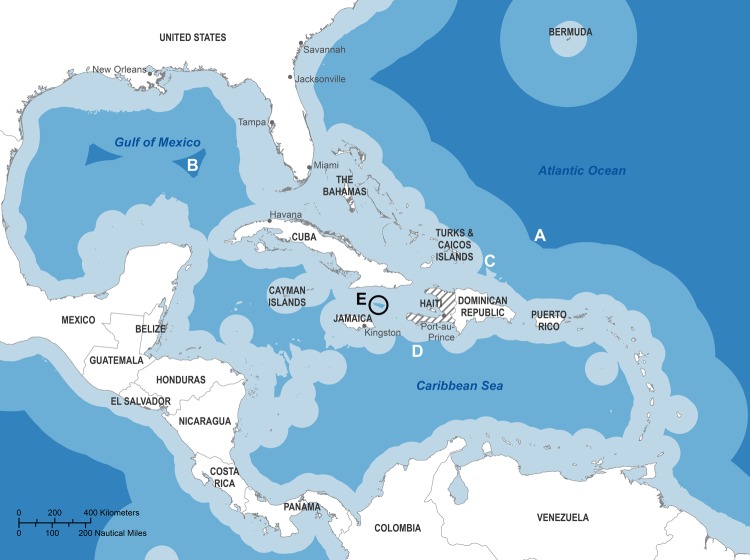
Zones in the Caribbean region where distance from shore and water depth meet International Maritime Organization guidelines for ballast exchange. To exchange ballast >200 nautical miles from shore in water 200 m deep, ships must travel 280 nautical miles northeast of Haiti (A) or to the Gulf of Mexico (B). To exchange ballast at the minimum 50 nautical miles from shore in water >200 m deep, ships must travel >90 nautical miles northeast (C) or 50 nautical miles south (D) of Haiti or conduct the exchange in an area <45 nautical miles wide approximately equidistant from Haiti, Cuba, and Jamaica (E). Light blue shading indicates distance from land is <50 nautical miles and/or seawater depth is <200 m. Medium blue shading indicates distance from land is >50 nautical miles but <200 nautical miles, and seawater depth is >200 m. Dark blue shading indicates distance from land is >200 nautical miles and seawater depth is >200 m.

After discussions with staff at CDC and the Pan American Health Organization, the Director General of the Haitian Ministry of Health and Population issued 2 memoranda addressing ballast water management. The first, issued November 10, 2011, asked ship captains not to exchange ballast water in Haitian harbors. The second, issued November 15, 2011, reminded ship captains to adhere to IMO guidelines for ballast water exchange. However, ships operating in the Caribbean Sea face practical difficulties in conducting ballast water exchange at the recommended distances without making large deviations from usual routes. The BWM Convention recommends that designated ballast water exchange areas should be on or close to existing maritime routes and does not require that ships deviate course or delay voyages to comply with ballast water exchange guidelines (*6*). Ballast water exchange at sea also presents operational and safety challenges to ships and might not be completely effective in preventing spread of aquatic organisms (*12*).

As an alternative management measure, ballast water treatment systems are being developed to meet the BWM Convention’s numeric discharge standards, and several systems are available (*13*). These systems combine filtration with nonchemical (e.g., UV light, shear, heat) and chemical biocides to remove or kill organisms (*14*). Ballast water treatment systems are designed to achieve the BWM Convention efficacy levels, and their future use in the Caribbean region would likely be a more effective management approach than ballast water exchange.

The Haiti cholera outbreak spread to the neighboring Dominican Republic, and cholera cases associated with travel to Haiti were recognized in the United States (*7*), but there is no evidence that *V. cholerae* was transferred by ship ballast water in these instances. The Wider Caribbean Region comprises 28 island and continental countries with coasts on the Caribbean Sea, the Gulf of Mexico, and adjacent waters of the Atlantic Ocean. This area is at elevated risk for transfer of contaminated ballast water because of the high volume of cargo trade in the region and has been prioritized by the Global Ballast Water Management Program (http://globallast.imo.org/index.asp), a program administered by IMO, the Global Environment Facility, and the United Nations Development Program to assist developing countries implement ballast water management measures (*15*).

## Conclusions

A comprehensive regional strategic plan has been developed to promote ratification of the BWM Convention and to facilitate its implementation within the Wider Caribbean Region through regional cooperation, training, communication, compliance monitoring and enforcement, and promotion of national-level legislation, task forces, action plans, and sustainable resources to support activities (*15*). Implementation of this plan will help protect public health by reducing the likelihood that *V. cholerae* and other pathogens will be transferred by ballast water.
